# Sex-disaggregated population analysis in patients with hidradenitis suppurativa

**DOI:** 10.3389/fmed.2022.1028943

**Published:** 2022-11-01

**Authors:** Robert Sabat, Athanasia Tsaousi, Kamran Ghoreschi, Kerstin Wolk, Sylke Schneider-Burrus

**Affiliations:** ^1^Interdisciplinary Group of Molecular Immunopathology, Dermatology/Medical Immunology, Charité—Universitätsmedizin Berlin, Corporate Member of Freie Universität Berlin and Humboldt- Universität zu Berlin, Berlin, Germany; ^2^Psoriasis Research and Treatment Center, Charité—Universitätsmedizin Berlin, Corporate Member of Freie Universität Berlin and Humboldt- Universität zu Berlin, Berlin, Germany; ^3^Department of Dermatology, Venereology and Allergology, Charité—Universitätsmedizin Berlin, Corporate Member of Freie Universität Berlin and Humboldt- Universität zu Berlin, Berlin, Germany; ^4^Institute of Medical Immunology, Charité—Universitätsmedizin Berlin, Corporate Member of Freie Universität Berlin and Humboldt- Universität zu Berlin, Berlin, Germany; ^5^Center for Dermatosurgery, Havelklinik Berlin, Berlin, Germany

**Keywords:** acne inversa, sex, obesity, smoking, metabolic syndrome, spondyloarthritis, family history, acne vulgaris

## Abstract

**Background:**

Hidradenitis suppurativa (HS) is a common chronic inflammatory skin disease, which affects both sexes.

**Objectives:**

Identification of sex-specific risk factors, comorbidity, clinical manifestations, and treatments in HS patients.

**Methods:**

A non-interventional, cross-sectional, mono-centric study with 500 HS patients. All patients were examined by dermatologists. Prospectively collected demographic, anamnestic, clinical data, and blood parameters were evaluated.

**Results:**

There were no significant differences in age at HS onset and in disease duration between female and male patients. Furthermore, no differences regarding the family history for HS were found between sexes. Regarding further risk factors for HS, central obesity was more frequent in women while extensive cigarette smoking and acne vulgaris were more commonly found among male patients. Regarding comorbidity, lower HDL-levels were significantly more frequent in men. Female patients were found to suffer significantly more often from back pain, especially in the neck/shoulder region and lower back. Analyzing the clinical manifestation of HS, the groin was more frequently involved in women and the axillae in men. Women showed a higher number of skin sites with inflammatory nodules, whereas fistulas were observed more frequently in men. Nevertheless, there was no difference in HS treatment applied to female vs. male patients.

**Limitations:**

Data were obtained from a mono-centric study.

**Conclusion:**

Significant differences in HS risk factors, comorbidity, and clinical manifestation exist between female and male patients. Thus, sex-specific differences should be taken into account in the prevention as well as medical and surgical treatment of HS patients.

## Introduction

Hidradenitis suppurativa (HS; also referred to as acne inversa) is a chronically relapsing inflammatory skin disease. It commonly affects the intertriginous skin of the axillary, inguinal, gluteal, and perianal sites and leads to excessive destruction of skin architecture (1). The clinical manifestation varies from recurring inflammatory nodules and abscesses to draining fistulas and extensive scars.

HS is recognized as a frequent disease, affecting ~1% of the general population with common onset in young adulthood (2–4). The painful, compromising skin lesions, large amounts of malodorous secretion, and disfigurement lead to a profound emotional and physical impact on HS-affected individuals, resulting in isolation, fear due to stigmatization in work and personal life, depression, and a severe quality of life impairment (5–9). HS has even a negative impact on the quality of life of family members of HS patients (10). HS patients frequently suffer from metabolic alterations including hypertriglyceridemia, hypo-HDL-cholesterolemia, hyperglycemia, and central obesity that may increase the risk of cardiovascular disorders and reduce life expectancy (11–13). Furthermore, back pain and spondyloarthritis often accompany HS (14, 15).

The pathogenesis of HS is only partially understood (16). A special combination of immune mediators in the skin lesions leads to the activation of immune and tissue cells and ultimately to the destruction of the normal skin architecture (17–23). From the etiological point of view, genetic predisposition and lifestyle factors contribute to the onset of the disease (16, 24, 25). Furthermore, the beginning of the disease after puberty, pre-menstrual flare-up, and improvement during pregnancy suggest a contribution of endocrinological factors in the development of the disease (26–29). Nevertheless, the levels of sex hormones are generally not elevated in patients with HS (30). Rather, initial data suggests that the number of androgen receptor-positive keratinocytes is increased in the epidermis of HS lesions (31). Accordingly, the results of RNA microarray analyses comparing HS lesions vs. non-lesional skin show an enrichment of molecules regulated by androgen receptors or transcription factors associated with epidermal stem cells (32). Furthermore, androgen receptor activation might elevate the TNF-α expression by myeloid cells *via* multiple mechanisms (33). Both women and men can be affected by HS (1). However, an earlier disease onset in women and a more severe disease course in men have been reported (34, 35). Information on further sex-related differences in patients with HS is lacking.

In this study, we examined possible sex-related risk factors and comorbidity in HS patients. Moreover, we aimed to identify sex-specific differences in clinical disease manifestation as well as in applied therapies, which may help to develop sex-specific patient care regimens.

## Materials and methods

### Patients

A non-interventional, cross-sectional, mono-centric study with 500 HS patients was conducted (9). The patients (i) visited the Department of Dermatology of the University Hospital Charité, Berlin, Germany, from February 2012 to November 2017, (ii) gave written informed consent, and (iii) fulfilled the following inclusion criteria: age of at least 18 years and diagnosed with HS (9). The diagnosis of HS was made by an experienced dermatologist based on generally accepted diagnostic criteria (the nature and localization of skin lesions and the disease course) (1). There were no specific exclusion criteria.

The study was conducted according to the principles expressed in the Declaration of Helsinki. Written informed consent was obtained from all participants. The study was approved by the clinical institutional review board (Ethikkommission) of Charité University Hospital (Charité - Universitätsmedizin Berlin), Berlin, Germany.

### Assessments

The data were collected at one point of time, and no follow-up visits were scheduled. The demographic characteristics, family history (FH), details of the course of HS (e.g., age at onset, disease duration), and information regarding coexisting conditions including acne vulgaris were collected using a questionnaire. Moreover, we asked for previous HS therapies including antibiotics or surgical therapy such as lancing of abscesses and excision of fistulas. Height, weight, waist circumference, clinical data (e.g., blood pressure), details of affected regions, hematology and clinical chemistry results (e.g., HDL-cholesterol, glucose) were assessed by a physician. Due to the non-interventional setting of the study, oral glucose tolerance test was not carried out. Central obesity was defined as waist circumference of ≥88 cm in female and ≥102 in male patients. Patients were referred to as positive for arterial hypertension at a systolic blood pressure of ≥130 or diastolic blood pressure of ≥85 mmHg, as well as when arterial hypertension or use of medication against hypertension were reported. Disease severity was assessed by the Sartorius score and the Hurley‘s three-degree scale. A higher score indicates greater severity of disease. The impairment in quality of life due to HS was assessed using the Dermatology Life Quality Index (DLQI). A higher score indicates profounder impairment.

### Statistical analysis

Statistical calculations were performed using Statistical Package for Social Science software (IBM, Ehningen, Germany). The aim of this manuscript - to identify possible sex-specific differences in the clinical manifestation of HS and in the applied therapies - was of exploratory nature. Thus, no power calculation was performed in advance. Continuous variables were described as means ±standard deviation (SD) or standard error of the mean (SEM) as indicated. Mann-Whitey-U test (two-tailed) was used for analyzing differences in continuous variables between female and male patients. Discontinuous variables were described by the total frequencies and percentages of each modality and were analyzed using the Chi-square test. Correlation analyses were performed using the Spearman's rank correlation test. Neither a multicollinearity testing nor multiple regression analyses were performed. Missing data were not replaced for analysis. The number of patients that gave information about specific parameters is indicated in the figure legends. Statistical significance was achieved if *P*-values were < 0.05 (^*^P < 0.05, ^**^ *P* ≤ 0.01, ^***^ *P* ≤ 0.001).

## Results

Five hundred patients suffering from HS (303 women and 197 men) were included in the study. The most important characteristics of the study population are displayed in Table 1. The mean ±SD age at onset of disease of female and male patients participating in the study was 25.9 ±1.1 and 24.9 ±9.8 (*P* = 0.570). The mean ±SD duration of disease of female and male HS patients was 13.8 ±10.0 and 12.7 ±9.4 (*P* = 0.293). Thus, no significant differences were found between the two groups with regard to the above-mentioned characteristics, allowing us to subsequently perform sex-disaggregated analyses.

**Table 1:** Demographic and clinical characteristics of the study cohort (*n* = 500).

	**HS patients**
**Age in years** (mean ± SD) (range)	38.9 ± 10.9 18.0−78,4
**Sex distribution**	
Females (%) Males (%)	60.6 39.4
**BMI** (mean ± SD) (range)	28.9 ± 5.9 17.2−52.6
**Smoking habit**	
Smokers (%)	66.6
Ex-smokers (%)	18.6
Never smokers (%)	14.8
**Disease duration, years** (mean ± SD) (range)	13.3 ± 9.8 0.1−52.9
**Hurley score** (mean ± SD) (range)	1.64 ± 0.89 0−3
**Sartorius score** (mean ± SD) (range)	49.3 ± 34.7 0−216
**Family history of HS** Positive (%) Negative (%)	33.8 66.2
**Patients reporting previous**: Abscess incision (%) Wide skin resection (%) Antibiotic treatment (%)	57.3 61.6 64.9

BMI, body mass index; HS, hidradenitis suppurativa; SD, standard deviation.

### Risk factors for HS

**Figure 1:**
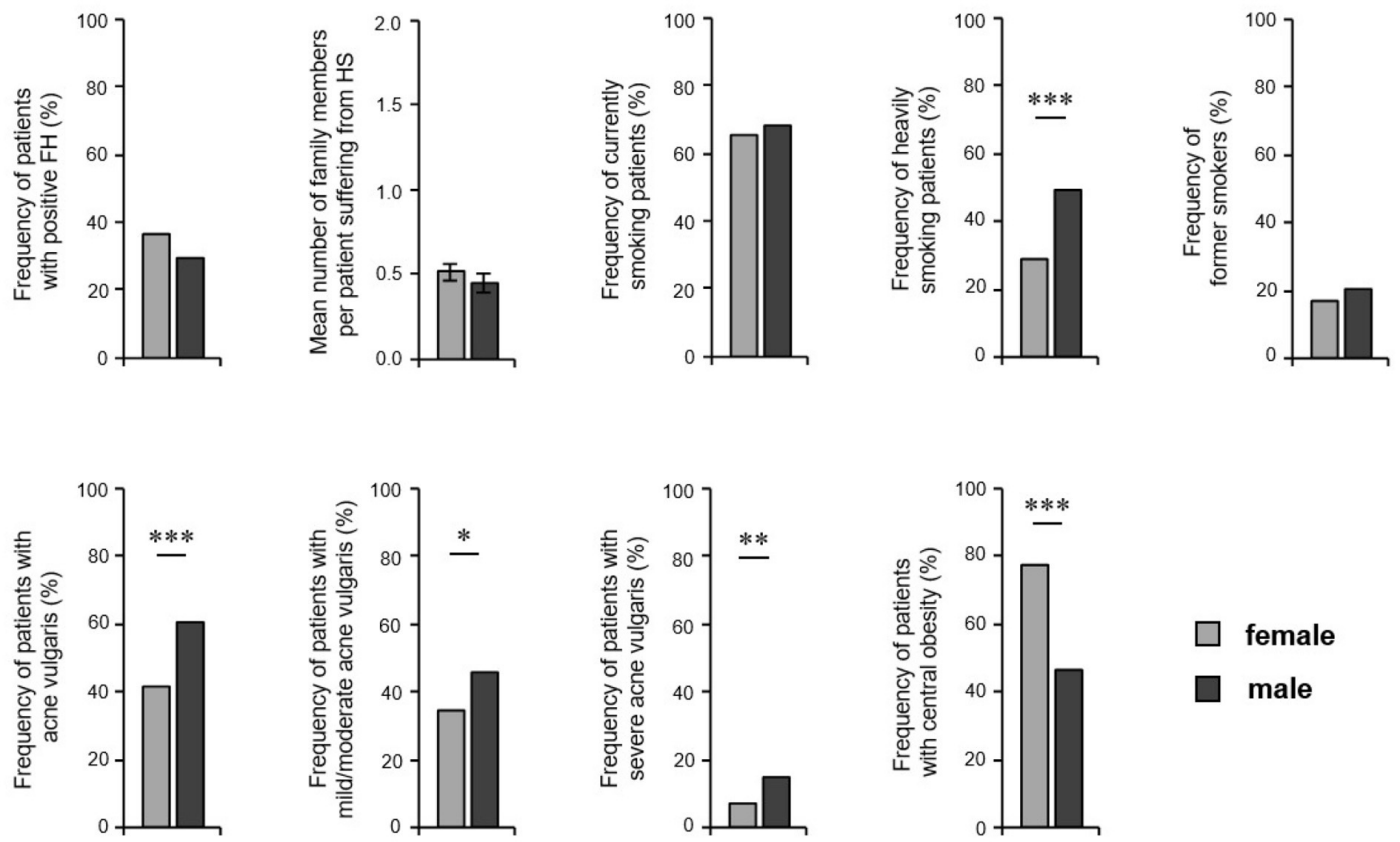
Risk factors for the development of HS in both sexes. The frequency of HS patients with positive family history (*n* = 473, female: 285, male: 188), the number of affected family members (*n* = 472, female: 284, male: 188), the frequency of currently smoking patients (*n* = 473, female: 284, male: 189) and those smoking an average number of cigarettes of >20 per day (*n* = 427, female: 261, male: 166), of former smokers (*n* = 472, female: 284, male: 188), as well as the frequency of patients with concomitant acne vulgaris (*n* = 442, female: 266, male: 176), and patients with central obesity (*n* = 385, female: 238, male: 147) are given. Significance of differences was assessed by the Chi-square test (* *P* < 0.05, ** *P* ≤ 0.01, *** *P* ≤ 0.001).

First, we analyzed the distribution of known HS risk factors (1), such as positive FH for HS, obesity, and acne vulgaris. The frequency of patients with positive FH did not differ significantly between female and male HS patients (Figure 1). Furthermore, the average number of family members suffering from HS was similar in both groups (0.51 for female and 0.45 for male HS patients; *P* = 0.258). However, male patients were significantly more often heavy smokers (>20 cigarettes per day; 29% women, 49% men; *P* < 0.001) and stated acne vulgaris in the self-reported history (41% women, 60% men; *P* < 0.001), whereas women showed significantly more frequently central obesity (77% women, 46% men; *P* < 0.001) (Figure 1). No significant differences were found between the sexes regarding the frequency of current or past smoking (Figure 1).

### Skin areas affected by HS

**Figure 2:**
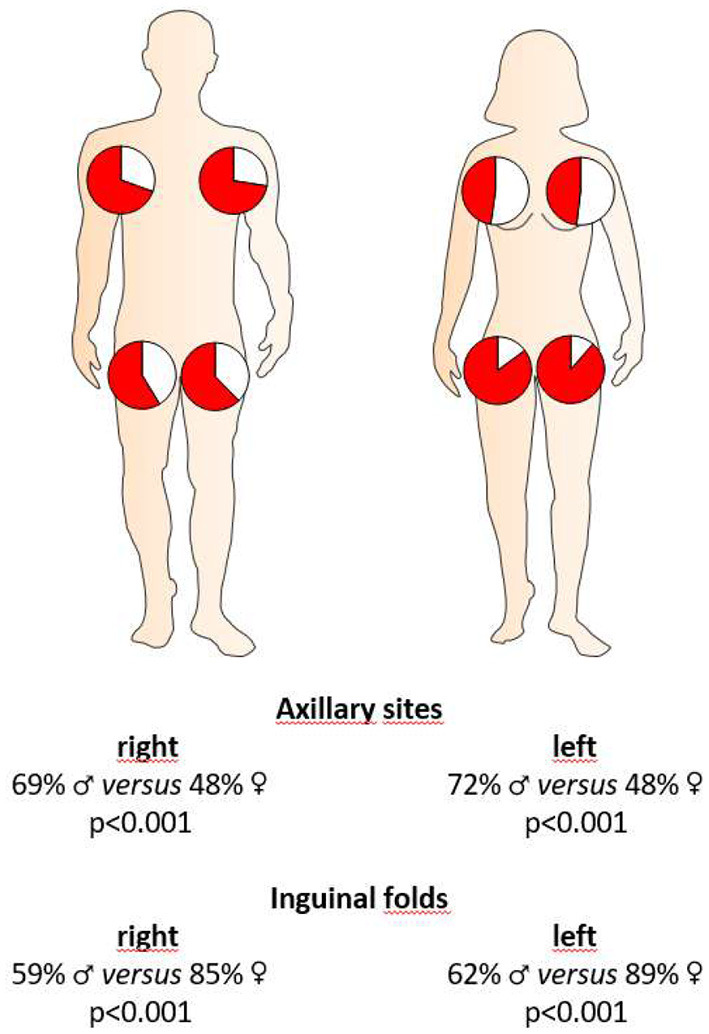
Localization of HS skin lesions in both sexes at the time of clinical presentation. Red coloration in the pie charts accounts for the percentage of female or male HS patients with respective localization of skin alterations. Significance of differences was assessed by the Chi-square test.

We further examined, whether there were any sex-specific differences in terms of the sites of clinical manifestation. Interestingly, in female HS patients, inguinal sites were significantly more frequently affected upon disease onset (37% for women, 20% for men; *P* < 0.001) and clinical examination upon enrollment in this study (Figure 2), whereas axillary sites were significantly more often affected in men upon disease outbreak (32% for women, 47% for men; *P* = 0.001) and enrollment (Figure 2).

### Severity of HS and clinical manifestation

**Figure 3:**
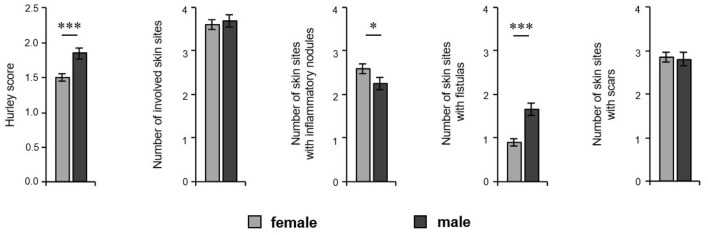
Extent of HS cutaneous alterations in both sexes. The disease severity (Hurley score, *n* = 424, female: 259, male: 163), the number of involved skin sites considering right and left axillary, right and left inguinal, and right and left gluteal sites as well as the pilonidal sinus, and the number of those skin regions with inflammatory nodules, fistulas, and scars of HS patients (*n* = 429, female: 262, male: 169) are demonstrated as the mean ±SEM. Significance of differences was assessed by the Mann–Whitney U-test (two-tailed; *P < 0.05, ***P ≤ 0.001).

In order to analyze a possible association between sex and disease severity, we documented the Hurley score and Sartorius score. Furthermore, we also documented the potential involvement of right and left axillary, inguinal, and gluteal areas as well as pilonidal sinus. Moreover, we counted those areas that contained nodules, fistulas and/or scars. Our study population mainly includes patients with mild-to-severe HS (Table 1). The disease severity, as assessed by the Hurley score, was significantly higher in male patients (1.51 for women, 1.84 for men; *P* < 0.001) (Figure 3). Using the Sartorius score, we found a non-significant difference between the sexes, with men tending to be more affected than women (46.6 for women, 53.6 for men; *P* = 0.088). However, the number of affected skin areas of interest was similar in both groups (Figure 3). Looking at the lesions in detail, male patients had a significantly greater number of areas with fistulas (0.91 for women, 1.66 for men; *P* < 0.001) compared to female participants, who were found with a higher number of areas with inflammatory nodules (2.58 for women, 2.25 for men; *P* = 0.047) (Figure 3). There was no significant difference in the number of skin sites with scars (Figure 3). Interestingly, the blood leukocyte count—as indicator for inflammation—was identical in female and male patients (mean ±SD counts/nl: 9.3 ±2.7 for women and 9.3 ±2.6 for men; *P* = 0.861) suggesting similar extent of inflammation in both groups. In addition, the impairment in quality of life, as assessed using the DLQI, was similar in female and male patients (mean ±SD: 13.6 ±7.8 for women and 12.6 ±8.2 for men; *P* = 0.181). Interestingly, there was a positive correlation between DLQI-values and blood leukocyte counts in both female and male patients (r_s_= 0.300; *P* = 0.003 for women and r_s_= 0.244; *P* = 0.023 for men).

### HS comorbidity

**Figure 4:**
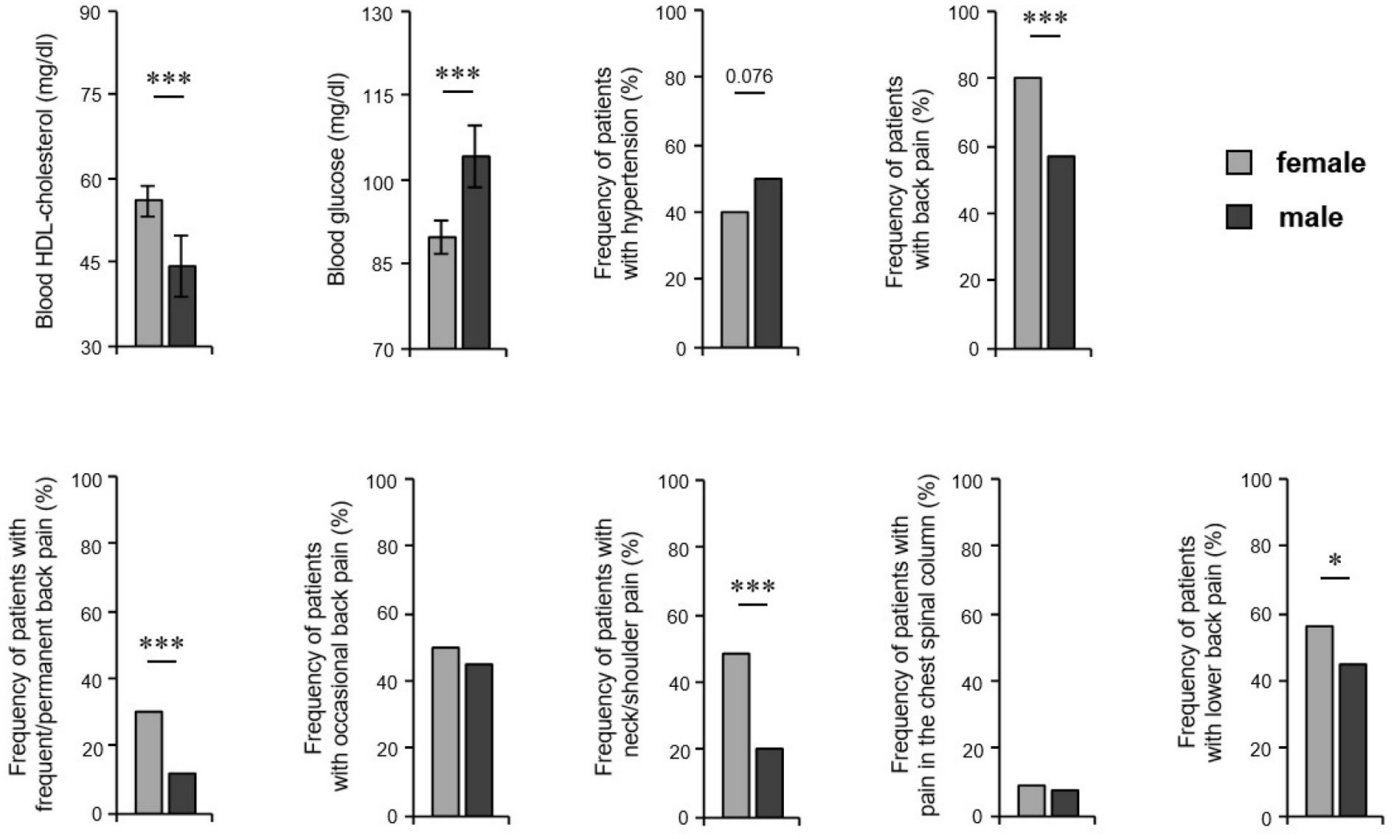
HS comorbidity in both sexes. HS patients' blood parameters including HDL-cholesterol (*n* = 110, female: 52, male: 58) and glucose (*n* = 122, female: 62, male: 60) are demonstrated as the mean ±SEM. The frequency of HS patients with arterial hypertension (*n* = 355, female: 220, male: 135) and back pain (overall and broken down into different pain frequency and localizations) (*n* = 474, female: 285, male: 189) are given. Significance of differences was assessed by the Mann–Whitney U-test (blood parameters) and the Chi-square test (pain frequency) (*P < 0.05, ***P ≤ 0.001).

We further asked whether the prevalence of the most common comorbid disorders differs between the sexes. Male patients had lower levels of blood HDL-cholesterol (56.0 mg/dL in women, 44.3 mg/dL in men; *P* < 0.001) and higher levels of glucose in blood (89.8 mg/dL in women, 104.3 mg/dL in men; *P* = 0.001) and showed a trend to arterial hypertension compared to female patients (40% of women, 50% of men; *P* = 0.076) (Figure 4). However, our analyses revealed that female patients suffered significantly more frequently from back pain (80% women, 57% men; *P* < 0.001), in particular permanent pain (30% of women, 12% of men; *P* < 0.000) (Figure 4). Moreover, women complained more often about pain in the neck/shoulder regions (49% of women, 20% of men; *P* < 0.001) and lower back (56% women, 45% men; *P* = 0.017) compared to male patients (Figure 4). Regarding Crohn's disease, we found no significant difference between the sexes (*P* = 0.267), which might be due to the low overall frequency of Crohn's disease and the limited number of patients included in this study.

### Treatment of HS

**Figure 5:**
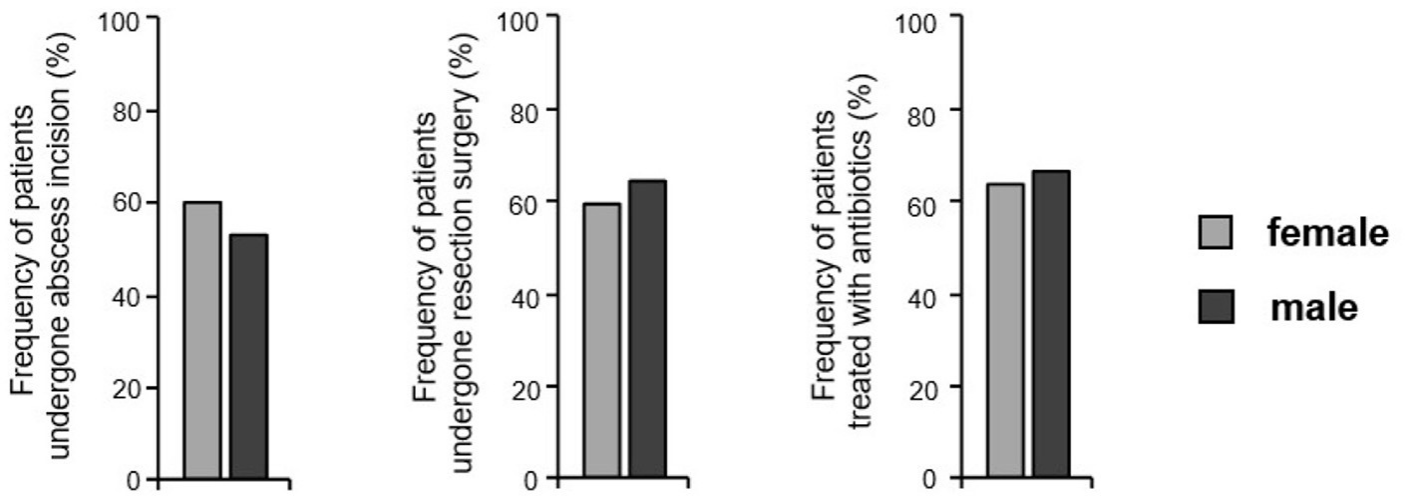
Treatments previously performed in both sexes. The frequency of HS patients reporting previous abscess incision, wide skin resection, and antibiotic treatment is given (*n* = 445, female: 268, male: 177). Significance of differences was assessed by the Chi-square test.

Finally, we wanted to find out, whether the differences in the localization and kind of cutaneous HS alterations and in the systemic comorbidity were associated with the use of different therapies in the past in HS patients. Surprisingly, no significant sex-specific differences could be detected in the proportion of patients who had undergone abscess incision, wide skin excision, or antibiotic treatment before enrollment in this study (Figure 5). It should be noted that the data described above, such as the Sartorius score and DLQI score, suggest that treatments used in the past have not led to a long-lasting improvement in either female or male patients.

## Discussion

Our study revealed a number of sex-related differences for disease risk factors, clinical aspects, and comorbidity in patients suffering from HS.

The high prevalence of the metabolic syndrome and other cardiovascular risk factors in HS patients has been established previously (11, 12, 36), suggesting that the metabolic syndrome might be a predisposing condition for HS (1). Investigating sex-specific aspects in this study, we found that parameters of the metabolic syndrome differed between sexes: Female patients suffered significantly more often from central obesity than male patients, while male participants demonstrated more often lower blood HDL-cholesterol levels, higher blood glucose levels, and experienced arterial hypertension than HS afflicted women.

In the general population, central obesity is reported almost twice as frequently in women as in men (43.7 vs. 24.8%) (37). As expected, the overall prevalence rate of 65% for central obesity among HS patients found in this study was much higher than in the general population, with a significant female dominance. Considering that obesity is known to activate the innate immune system (38, 39), this high prevalence may indicate that obesity is one of the key factors that leads to development of this inflammatory skin disease in women. Interestingly, abdominal obesity was the most frequent aspect of the metabolic syndrome not only in female HS patients (this study) but also in female psoriasis patients (40). There is also a positive association between psoriasis severity and central obesity (40), underlining again the possible association of the amount of fat tissue and the degree of inflammation.

As published by the World Health Organization for high-income countries, women smoke at nearly the same rate as men (41). German data match this finding, suggesting that during the year 2015 26% of women and 31% of men were smokers (42). We found more than twice as high rates of smokers in our group of HS patients, with 65% of female and 68% of male HS patients being smokers. Considering heavy smokers with a consumption rate of >20 cigarettes per day, there is a pronounced sex-related difference in the general population: 4.3% of women and 8.0% of men are heavy smokers. In our HS patients, the overall percentage of heavy smokers was much higher; however, the ratio between men and women was similar (29% of women, 50% of men). It has been previously shown that HS is more severe in smokers than in non-smokers (43). Hence, the higher proportion of especially heavy smokers among male HS patients might help to explain why the prevalence of HS is only slightly lower for male compared to female individuals. In fact, two independent, recently published studies from Germany with larger numbers of HS patients have documented a sex distribution among HS patients with ~55% women vs. ~45% men (44, 45).

In contrast to central obesity and extensive cigarette smoking, we observed no sex-related differences regarding the FH for HS. This suggests that the genetic contribution for HS is similar in female and male patients.

We showed that the axillary skin sites were affected significantly more frequently in male than in female HS patients. In contrast, the groin was involved more frequently in women than in male HS patients. In fact, the groin was affected in almost 90% of female HS patients compared to 60% of men. In addition, our study disclosed that fistulas and, in consequence, Hurley stages II and III were significantly more often observed in men, whereas inflammatory nodules (often associated with Hurley stage I) affected female study subjects more commonly. Thus, this might explain the fact that male patients are often afflicted with higher Hurley stages (34). Importantly, taking into account the fact that female and male patients in our study reported a similar HS disease duration, the different nodule and fistula counts suggest sex-specific inflammation/ skin destruction in HS. However, this very exciting hypothesis needs to be verified in subsequent studies. Furthermore, studies examining patients at multiple time points are required to capture further possible differences in the progression of the disease between the sexes (e.g., relapse rate after treatments). In our study the data were collected at only one point of time, and no follow-up visits were scheduled, which therefore is a limitation.

Since the treatment of fistulas or inflammatory nodules should be different, we expected differences between the two sexes in the previous therapies, with more excisions in men and more conservative treatments in women. However, no significant differences in the therapeutic procedure, which the two groups had undergone, were reported. This may be due to the fact that the HS therapy algorithms are still being under development (1, 46–48).

Considering the sex differences detected with regard to the above-mentioned disease risk factors and comorbidity, we advocate a more sex-related approach when it comes to patient management. In particular, we suggest more sex-specific life-style measures, e.g., smoking cessation in male and weight reduction or increase of physical activity in female HS patients. In this regard, a frequent monitoring of clinical parameters (e.g., blood pressure) and clinical chemistry (e.g., HDL-cholesterol) should be an indestructible part of the medical consultation for both sexes. Hence, we suggest that these sex-specific differences should be taken into consideration when caring for patients with HS.

## Data availability statement

The main data are presented within tables and figures of the manuscript. Further data will be made available upon request according to the legal and ethical possibilities by the corresponding author.

## Ethics statement

The study was conducted according to the principles expressed in the Declaration of Helsinki. Written informed consent was obtained from all participants. The study was approved by the clinical institutional review board (Ethikkommission) of Charité University Hospital (Charité - Universitätsmedizin Berlin), Berlin, Germany.

## Author contributions

RS: conceptual idea of the manuscript, statistical analysis, and drafting and revision of the manuscript. AT: data collection, contribution to data curation, and revision of the manuscript. KG: data analysis and revision of the manuscript. KW: data analysis, visualization of the results, and revision of the manuscript. SS-B: design of the study, conceptual idea of the manuscript, data collection and analysis, and drafting and revision of the manuscript. All authors contributed to the article and approved the submitted version.

## Conflict of interest

Author RS has received research grants or honoraria for participation in advisory boards, clinical trials, or as speaker for one or more of the following: AbbVie Inc., AbbVie Deutschland GmbH & Co., KG, Amgen GmbH, Bayer Schering Pharma AG, Biogen Idec GmbH, Boehringer Ingelheim Pharma GmbH & Co., KG, Celgene GmbH, Celgene International II Sàrl, Charité Research Organisation GmbH, CSL Behring, Dr. Willmar Schwabe GmbH & Co., KG, Flexopharm GmbH & Co., KG, Incyte Corporation, JanssenCilag GmbH, La Roche-Posay Laboratoire Dermatologique, Novartis Pharma GmbH, Parexel International GmbH, Sanofi-Aventis Deutschland GmbH, TFS GmbH, and UCB Biopharma SPRL. Author KW has received research grants or honoraria for participation in advisory boards, clinical trials, or as speaker for one or more of the following: AbbVie Inc., AbbVie Deutschland GmbH & Co., KG, Celgene/BMS, Charité Research Organisation GmbH, Dr. Willmar Schwabe GmbH & Co., KG, Flexopharm GmbH & Co., KG, JanssenCilag GmbH, Novartis Pharma GmbH, Pfizer Deutschland GmbH, Sanofi-Aventis Deutschland GmbH, TFS GmbH, and UCB Biopharma SPRL. Author SS-B has received research grants or honoraria for participation in advisory boards, clinical trials, or as speaker for one or more of the following: AbbVie Inc., AbbVie Deutschland GmbH & Co., KG, Biogen Idec GmbH, Moonlake Immunotherapeutics, Novartis Pharma GmbH, Parexel International GmbH, UCB Biopharma SPRL. The remaining authors declare that the research was conducted in the absence of any commercial or financial relationships that could be construed as a potential conflict of interest.

## Publisher's note

All claims expressed in this article are solely those of the authors and do not necessarily represent those of their affiliated organizations, or those of the publisher, the editors and the reviewers. Any product that may be evaluated in this article, or claim that may be made by its manufacturer, is not guaranteed or endorsed by the publisher.

## Abbreviations

FH: family history
HDL: high-density lipoprotein
HS: hidradenitis suppurativa
SD: standard deviation
SEM: standard error of the mean.

## References

[1] Sabat R Jemec GBE Matusiak L Kimball AB Prens E Wolk K. Hidradenitis suppurativa. Nat Rev Dis Primers. (2020) 6:18. 10.1038/s41572-020-0149-1 32165620

[2] Jemec GB Heidenheim M Nielsen NH. The prevalence of hidradenitis suppurativa and its potential precursor lesions. J Am Acad Dermatol. (1996) 35:191–4. 10.1016/S0190-9622(96)90321-7 8708018

[3] Ingram JR. The epidemiology of hidradenitis suppurativa. Br J Dermatol. (2020) 183:990–8. 10.1111/bjd.19435 32880911

[4] Sachdeva M Shah M Alavi A. Race-specific prevalence of hidradenitis suppurativa. J Cutan Med Surg. (2021) 25:177–87. 10.1177/1203475420972348 33174482

[5] Wolkenstein P Loundou A Barrau K Auquier P Revuz J Quality of Life Group of the French Society of Dermatology. Quality of life impairment in hidradenitis suppurativa: a study of 61 cases. J Am Acad Dermatol. (2007) 56:621–3. 10.1016/j.jaad.2006.08.061 17097366

[6] Matusiak L Bieniek A Szepietowski JC. Psychophysical aspects of hidradenitis suppurativa. Acta Derm Venereol. (2010) 90:264–8. 10.2340/00015555-0866 20526543

[7] Kurek A Peters EM Chanwangpong A Sabat R Sterry W Schneider-Burrus S. Profound disturbances of sexual health in patients with acne inversa. J Am Acad Dermatol. (2012) 67, 422–8. 10.1016/j.jaad.2011.10.024 22182915

[8] Kurek A Johanne Peters EM Sabat R Sterry W Schneider-Burrus S. Depression is a frequent co-morbidity in patients with acne inversa. J Dtsch Dermatol Ges. (2013) 11, 743–9. 10.1111/ddg.12067 23565584

[9] Schneider-Burrus S Tsaousi A Barbus S Huss-Marp J Witte K Wolk K. Features associated with quality of life impairment in hidradenitis suppurativa patients. Front Med (Lausanne). (2021) 8:676241. 10.3389/fmed.2021.676241 33987196 PMC8112201

[10] Wlodarek K Glowaczewska A Matusiak L Szepietowski JC. Psychosocial burden of Hidradenitis Suppurativa patients' partners. J Eur Acad Dermatol Venereol. (2020) 34:1822–7. 10.1111/jdv.16255 32003871

[11] Sabat R Chanwangpong A Schneider-Burrus S Metternich D Kokolakis G Kurek A. Increased prevalence of metabolic syndrome in patients with acne inversa. PLoS ONE. (2012) 7:e31810. 10.1371/journal.pone.0031810 22359634 PMC3281019

[12] Hung CT Chiang CP Chung CH Tsao CH Chien WC Wang WM. Increased risk of cardiovascular comorbidities in hidradenitis suppurativa: A nationwide, population-based, cohort study in Taiwan. J Dermatol. (2019) 46:867–73. 10.1111/1346-8138.15038 31389066

[13] Tiri H Jokelainen J Timonen M Tasanen K Huilaja L. Substantially reduced life expectancy in patients with hidradenitis suppurativa: a Finnish nationwide registry study. Br J Dermatol. (2019) 180:1543–4. 10.1111/bjd.17578 30597518

[14] Schneider-Burrus S Witte-Haendel E Christou D Rigoni B Sabat R Diederichs G. High prevalence of back pain and axial spondyloarthropathy in patients with hidradenitis suppurativa. Dermatology. (2016) 232:606–12. 10.1159/000448838 27649417

[15] Almuhanna N Finstrad A Alhusayen R. Association between hidradenitis suppurativa and inflammatory arthritis: a systematic review and meta-analysis. Dermatology. (2021) 1–8. 10.1159/000514582 33774640

[16] Wolk K Join-Lambert O Sabat R. Aetiology and pathogenesis of hidradenitis suppurativa. Br J Dermatol. (2020) 183:999–1010. 10.1111/bjd.19556 33048349

[17] Wolk K Warszawska K Hoeflich C Witte E Schneider-Burrus S Witte K. Deficiency of IL-22 contributes to a chronic inflammatory disease: pathogenetic mechanisms in acne inversa. J Immunol. (2011) 186:1228–39. 10.4049/jimmunol.0903907 21148041

[18] Caprio RDi Balato A Caiazzo G Lembo S Raimondo A Fabbrocini G. IL-36 cytokines are increased in acne and hidradenitis suppurativa. Arch Dermatol Res. (2017) 309:673–8. 10.1007/s00403-017-1769-5 28852851

[19] Hessam S Sand M Gambichler T Skrygan M Ruddel I Bechara FG. Interleukin-36 in hidradenitis suppurativa: evidence for a distinctive proinflammatory role and a key factor in the development of an inflammatory loop. Br J Dermatol. (2018) 178:761–7. 10.1111/bjd.16019 28975626

[20] Witte-Händel E Wolk K Tsaousi A Irmer ML Mößner R Shomroni O. The IL-1 pathway is hyperactive in hidradenitis suppurativa and contributes to skin infiltration and destruction. J Invest Dermatol. (2019) 139:1294–305. 10.1016/j.jid.2018.11.018 30528824

[21] Scala E Caprio RDi Cacciapuoti S Caiazzo G Fusco A Tortorella E. A new T helper 17 cytokine in hidradenitis suppurativa: antimicrobial and proinflammatory role of interleukin-26. Br J Dermatol. (2019) 181:1038–45. 10.1111/bjd.17854 30829398

[22] Batycka-Baran A Baran W Nowicka-Suszko D Koziol-Gałczyńska M Bieniek A Matusiak Ł. (2020). Serum Concentration and Skin Expression of S100A7 (Psoriasin) in Patients Suffering from Hidradenitis Suppurativa. Dermatology (2020) 1-7. 10.1159/000510689 33202403

[23] Wolk K Brembach TC Simaite D Bartnik E Cucinotta S Pokrywka A. Activity and components of the granulocyte colony-stimulating factor pathway in hidradenitis suppurativa. Br J Dermatol. (2021) 185:164–76. 10.1111/bjd.19795 33400270

[24] van Straalen KR Prens EP Willemsen G Boomsma DI van der Zee HH. Contribution of genetics to the susceptibility to hidradenitis suppurativa in a large, cross-sectional dutch twin cohort. JAMA Dermatol. (2020) 156:1359–62. 10.1001/jamadermatol.2020.3630 33052394 PMC7557497

[25] Hessam S Gambichler T Skrygan M Scholl L Sand M Meyer T. Increased expression profile of NCSTN, Notch and PI3K/AKT3 in hidradenitis suppurativa. J Eur Acad Dermatol Venereol. (2021) 35:203–10. 10.1111/jdv.16962 32978818

[26] Barth JH Layton AM Cunliffe WJ. Endocrine factors in pre- and postmenopausal women with hidradenitis suppurativa. Br J Dermatol. (1996) 134:1057–9. 10.1111/j.1365-2133.1996.tb07942.x 8763424

[27] Vossen AR van Straalen KR Prens EP van der Zee HH. Menses and pregnancy affect symptoms in hidradenitis suppurativa: A cross-sectional study. J Am Acad Dermatol. (2017) 76:155–6. 10.1016/j.jaad.2016.07.024 27986138

[28] Karagiannidis I Nikolakis G Sabat R Zouboulis CC. Hidradenitis suppurativa/Acne inversa: an endocrine skin disorder? Rev Endocr Metab Disord. (2016) 17:335–41. 10.1007/s11154-016-9366-z 27294593

[29] Lyons AB Peacock A McKenzie SA Jacobsen G Naik HB Shi VY. Evaluation of hidradenitis suppurativa disease course during pregnancy and postpartum. JAMA Dermatol. (2020) 156:681–5. 10.1001/jamadermatol.2020.0777 32347884 PMC7191431

[30] Riis PT Ring HC Themstrup L Jemec GB. The role of androgens and estrogens in hidradenitis suppurativa—a systematic review. Acta Dermatovenerol Croat. (2016) 24:239–49. 28128074

[31] Yu W Barrett J Liu P Parameswaran A Chiu ES Lu CP. Novel evidence of androgen receptor immunoreactivity in skin tunnels of hidradenitis suppurativa: assessment of sex and individual variability. Br J Dermatol. (2021) 185:855–8. 10.1111/bjd.20520 34047363

[32] Gauntner TD. Hormonal, stem cell and Notch signalling as possible mechanisms of disease in hidradenitis suppurativa: a systems-level transcriptomic analysis. Br J Dermatol. (2019) 180:203–4. 10.1111/bjd.17093 30117141

[33] Gratton R Del Vecchio C Zupin L Crovella S. Unraveling the role of sex hormones on keratinocyte functions in human inflammatory skin diseases. Int J Mol Sci. (2022) 23. 10.3390/ijms23063132 35328552 PMC8955788

[34] Schrader AM Deckers IE van der Zee HH Boer J Prens EP. Hidradenitis suppurativa: a retrospective study of 846 Dutch patients to identify factors associated with disease severity. J Am Acad Dermatol. (2014) 71:460–7. 10.1016/j.jaad.2014.04.001 24880664

[35] Naik HB Paul M Cohen SR Alavi A Suarez-Farinas M Lowes MA. Distribution of self-reported hidradenitis suppurativa age at onset. JAMA Dermatol. (2019) 155:971–3. 10.1001/jamadermatol.2019.0478 31166574 PMC6551578

[36] Reddy S Strunk A Jemec GBE Garg A. Incidence of myocardial infarction and cerebrovascular accident in patients with hidradenitis suppurativa. JAMA Dermatol. (2020) 156:65–71. 10.1001/jamadermatol.2019.3412 31721983 PMC6865223

[37] Kuk JL Ardern CI. Age and sex differences in the clustering of metabolic syndrome factors: association with mortality risk. Diabetes Care. (2010) 33:2457–61. 10.2337/dc10-0942 20699434 PMC2963512

[38] Wolk K Sabat R. Adipokines in psoriasis: An important link between skin inflammation and metabolic alterations. Rev Endocr Metab Disord. (2016) 17:305–17. 10.1007/s11154-016-9381-0 27554109

[39] Saltiel AR Olefsky JM. Inflammatory mechanisms linking obesity and metabolic disease. J Clin Invest. (2017) 127:1–4. 10.1172/JCI92035 28045402 PMC5199709

[40] Danielsen K Wilsgaard T Olsen AO Eggen AE Olsen K Cassano PA. Elevated odds of metabolic syndrome in psoriasis: a population-based study of age and sex differences. Br J Dermatol. (2015) 172:419–27. 10.1111/bjd.13288 25059341 PMC4338759

[41] WHO Report on the Global Tobacco Epidemic, 2008: The MPOWER Package. Geneva: World Health Organization (2008).

[42] Piontek D Gomes de Matos E Atzendorf J Kraus L. Kurzbericht Epidemiologischer Suchtsurvey 2015. Tabellenband: Tabakkonsum und Hinweise auf klinisch relevanten Tabakkonsum nach Geschlecht und Alter im Jahr 2015 (2016). München: IFT Institut für Therapieforschung.

[43] Sartorius K Emtestam L Jemec GB Lapins J. Objective scoring of hidradenitis suppurativa reflecting the role of tobacco smoking and obesity. Br J Dermatol. (2009) 161:831–9. 10.1111/j.1365-2133.2009.09198.x 19438453

[44] Kokolakis G Wolk K Schneider-Burrus S Kalus S Barbus S Gomis-Kleindienst S. Delayed diagnosis of hidradenitis suppurativa and its effect on patients and healthcare system. Dermatology. (2020) 236:421–30. 10.1159/000508787 32610312 PMC7592906

[45] Schneider-Burrus S Lux G van der Linde K Barbus S Huss-Marp J Tsaousi A. Hidradenitis suppurativa - prevalence analyses of German statutory health insurance data. J Eur Acad Dermatol Venereol. (2021) 35:e32–5. 10.1111/jdv.16783 32580237

[46] Alavi A Lynde C Alhusayen R Bourcier M Delorme I George R. Approach to the Management of Patients With Hidradenitis Suppurativa: A Consensus Document. J Cutan Med Surg. (2017) 21:513–24. 10.1177/1203475417716117 28639459

[47] Seyed Jafari SM Hunger RE Schlapbach C. Hidradenitis suppurativa: current understanding of pathogenic mechanisms and suggestion for treatment algorithm. Front Med (Lausanne). (2020) 7:68. 10.3389/fmed.2020.00068 32195261 PMC7064439

[48] Ujiie H Rosmarin D Schon MP Stander S Boch K Metz M. Unmet medical needs in chronic, non-communicable inflammatory skin diseases. Front Med (Lausanne). (2022) 9:875492. 10.3389/fmed.2022.875492 35755063 PMC9218547

